# Durable response to olaparib combined low-dose cisplatin in advanced hepatocellular carcinoma with FANCA mutation: A case report

**DOI:** 10.1097/MD.0000000000030719

**Published:** 2022-09-30

**Authors:** Yi-Hsuan Lai, Kai-Che Tung, San-Chi Chen

**Affiliations:** a Department of Medical Education, Taipei Veterans General Hospital, Taipei, Taiwan; b ACT Genomics Co., Ltd, Taipei, Taiwan; c Department of Medical Science and Institute of Bioinformatics and Structural Biology, National Tsing Hua University, Hsinchu, Taiwan; d School of Medicine, College of Medicine, National Yang Ming Chiao Tung University, Taipei, Taiwan; e Division of Medical Oncology, Center of Immuno-Oncology, Department of Oncology, Taipei Veterans General Hospital, Taipei, Taiwan; f Institute of Clinical Medicine, National Yang Ming Chiao Tung University, Taipei, Taiwan.

**Keywords:** case report, *FANCA*, hepatocellular carcinoma, homologous recombinational repair, olaparib, PARP inhibitor

## Abstract

To date, there is no actionable gene has been discovered in hepatocellular carcinoma (HCC). Tumor cells with DNA damage response and repair (DDR) gene loss-of-function mutation is sensitivity to poly-ADP-ribose polymerase (PARP) inhibitors and platinum chemotherapy in ovarian, prostate and pancreatic cancers. There is a case report demonstrated the efficacy of PARP inhibitor for *BRCA2* mutation that belongs to DDR gene in HCC, which suggested the potential role of PARP inhibitor for HCC with DDR gene mutation.

**Patient concerns:**

We reported a 44-year-old woman with non-viral HCC who was refractory to multiple treatment including target therapy, immunotherapy, and chemotherapy. The tumor tissue was submitted to next-generation sequencing using the commercially available ACTOnco®+ (ACT Genomics, Taiwan) assay that interrogates 440 and 31 cancer-related genes and fusion genes, respectively.

**Diagnosis:**

A truncating mutation *FANCA* p.Q1307fs was also observed. The tumor was microsatellite stable and had low tumor mutational burden of 4.5 muts/Mb.

**Interventions and outcomes:**

Given FANCA belongs to DDR genes, the inactivation evoked the idea of using PARP inhibitor and cisplatin. Therefore, the patient started to use olaparib combined with low-dose cisplatin (30 mg/m^2^, every 4 weeks) therapy in December 2019. Significant reduction in the tumor marker level in 1 month (PIVKA-II from 17,395 to 411 ng/dL) and follow-up CT scan showed stable disease. Her tumor did not progress until December 2020 with a progression-free survival of 12 months.

**Lessons:**

We report the first case of *FANCA*-mutated HCC that responded well to olaparib and low-dose cisplatin. This addressed the potential therapeutic role of DDR gene mutation in HCC and the possible synergistic effect of PARP inhibitor and cisplatin. These findings highlight areas where further investigation and effort are needed.

## 1. Introduction

Hepatocellular carcinoma (HCC) is the third leading cause of cancer-related death worldwide. The standard treatment includes multiple-kinase inhibitors and anti-PD-1/PD-L1. Recently, atezolizumab combined bevacizumab have been firstly recommended in advanced HCC due to the better efficacy than sorafenib. However, there is no actionable driven gene has been discovered in HCC. DNA damage response and repair (DDR) is responsible for homologous recombination repair), which is an important process when DNA double-strand breaks. Alterations of DDR genes lead to homologous recombination deficiency, genomic instability, and higher tumor mutational burden (TMB) in cancers. Common DDR genes include *BRCA1/2, PALB2, CDK12, RAD51, CHEK2, ATM*, and *FANCA*. Among these, BRCA1/2 are well-known to increase the cancer risk.^[1]^ The Fanconi anemia (FA) pathway, also called the FA-BRCA pathway, is an essential DNA repair pathway that recognized DNA damage and orchestrated DNA damage responses. The FA core complex that encoded by *FANCA* and *FANCG* interacts with other DNA-repair proteins to perform homologous recombination.^[2]^ On the contrary, the mutation of *FANCA* disrupts FA-BRCA repair pathway leads to the increase of sensitivity to DNA damaging agents.

In HCC, the incidence of DDR genes mutation is up to 20.9% and *BAP1*, *CHEK2* are the most common. Regarding to *FANCA* mutations, the incidence is only 2% of HCC patients.^[3]^ Poly-ADP-ribose polymerase (PARP) is a crucial protein in the DNA single-strand break repair pathway but also plays a role in double-strand break repair pathway. The inhibition of PARP leads to accumulation of unpaired single-strand break, which are converted to double-strand break. Thus, in the presence of DDR genes mutation, the use of PAPR inhibitor results in synthetic lethality in breast, ovarian, and prostate cancers.^[4–8]^ However, the efficacy of PARP inhibitor for DDR genes mutation in HCC is unknown. In this article, we present a patient of HCC with *FANCA* mutation who achieved durable response to the combination of olaparib and low-dose cisplatin.

## 2. Case presentation

In October 2017, a 44-year-old female was admitted to the Veteran General Hospital with right upper quadrant abdominal fullness. A tumor sized 15 cm with diaphragm invasion was found, without portal vein thrombosis. Patient was diagnosed with HCC, pT3aN0Mx, stage IIIA, and BCLC stage B. She underwent segmentectomy but 2 months later at least 4 tumors recurred. Thus, she underwent repeated trans-arterial chemoembolization but recurrence persisted. Due to trans-arterial chemoembolization refractory, she received systemic therapy subsequently, including lenvatinib, nivolumab, gemcitabine plus cisplatin and bevacizumab, doxorubicin, dacarbazine, FOLFOX (fluorouracil, oxaliplatin), and sorafenib. Her tumor did not respond to any of the above treatment and the tumor persisted to progress. Multiple bone metastasis and left humeral pathologic fracture developed in 2019 and thus she received open reduction internal fixation and palliative radiotherapy. Systemic treatment was changed to regorafenib, and pembrolizumab. However, her tumor still progressed.

**Figure 1.**
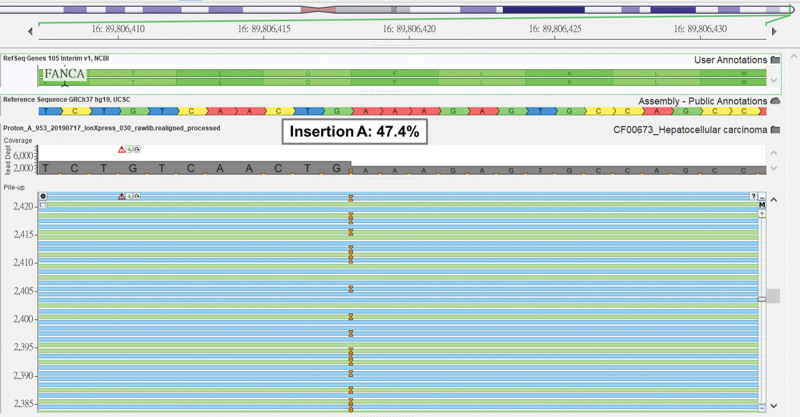
The next generation sequencing (NGS) results of the left humeral metastasis tumor biopsy. The NGS results showed the *FANCA* Q1307fs (c.3918dupT) that caused truncated loss-of-function *FANCA* protein.

The soft tissue from left humeral metastasis was subjected to next-generation sequencing (NGS) using the commercially available ACTOnco^®+^ (ACT Genomics, Taiwan) assay that interrogates 440 and 31 cancer-related genes and fusion genes, respectively (Tables 1 and 2). A total of 24 single nucleotide variants and small insertions and deletions were identified. Neither copy number amplification nor homozygous deletion was identified. The tumor was microsatellite stable and had low tumor mutational burden of 4.5 muts/Mb. Among these alterations, the *CTNNB1* p.K335I gain-of-function mutation was considered oncogenic but not actionable. A truncating mutation *FANCA* p.Q1307fs was also observed (Fig. 1).

**Table 1:** Gene contents of ACTOnco^®+^ assay. ACTOnco^®+^ assay identifies genetic alternations (single nucleotide variants, small insertions and deletions, and copy number variations) of 440 cancer-related genes, tumor mutational burden (TMB) and microsatellite instability (MSI) status from DNA.

ABCB1*	*AURKB*	*CBL*	*CDKN2B*	*E2F3*	*FAT1*	*GRIN2A*	*JAK2*	*MED12*	*NOTCH4*	*PMS1*	*RAD51D*	*SLCO1B3* *	*TNFRSF14*
ABCC2*	***AXIN1***	***CCNA1***	***CDKN2C***	***EGFR***	***FBXW7***	***GSK3B***	***JAK3***	***MEF2B***	***NPM1***	***PMS2***	***RAD52***	***SMAD2***	***TNFSF11***
ABCG2*	***AXIN2***	***CCNA2***	CEBPA*	***EP300***	***FCGR2B***	GSTP1*	***JUN***	***MEN1***	NQO1*	***POLB***	***RAD54L***	***SMAD3***	***TOP1***
*ABL1*	***AXL***	***CCNB1***	***CHEK1***	***EPCAM***	FGF1*	***GSTT1***	***KAT6A***	***MET***	***NRAS***	***POLD1***	***RAF1***	***SMAD4***	***TP53***
*ABL2*	***B2M***	***CCNB2***	***CHEK2***	***EPHA2***	***FGF10***	***HGF***	***KDM5A***	***MITF***	***NSD1***	***POLE***	***RARA***	***SMARCA4***	***TPMT***
*ADAMTS1*	***BAP1***	***CCNB3***	***CIC***	***EPHA3***	***FGF14***	***HIF1A***	***KDM5C***	***MLH1***	***NTRK1***	***PPARG***	***RB1***	***SMARCB1***	***TSC1***
*ADAMTS13*	***BARD1***	***CCND1***	***CREBBP***	***EPHA5***	***FGF19***	HIST1H1C*	***KDM6A***	***MPL***	***NTRK2***	***PPP2R1A***	***RBM10***	***SMO***	***TSC2***
*ADAMTS15*	***BCL10***	***CCND2***	***CRKL***	***EPHA7***	***FGF23***	HIST1H1E*	***KDR***	***MRE11***	***NTRK3***	***PRDM1***	***RECQL4***	SOCS1*	***TSHR***
*ADAMTS16*	***BCL2***	***CCND3***	***CRLF2***	***EPHB1***	***FGF3***	***HNF1A***	***KEAP1***	***MSH2***	***PAK3***	***PRKAR1A***	***REL***	***SOX2***	***TYMS***
*ADAMTS18*	***BCL2L1***	***CCNE1***	***CSF1R***	***ERBB2***	FGF4*	***HR***	***KIT***	***MSH6***	***PALB2***	***PRKCA***	***RET***	***SOX9***	***U2AF1***
*ADAMTS6*	***BCL2L2***	***CCNE2***	***CTCF***	***ERBB3***	***FGF6***	***HRAS***	***KMT2A***	***MTHFR*** *	***PARP1***	***PRKCB***	***RHOA***	***SPEN***	***UBE2A***
*ADAMTS9*	***BCL6***	***CCNH***	***CTLA4***	***ERBB4***	***FGFR1***	***HSP90AA1***	***KMT2C***	***MTOR***	***PAX5***	***PRKCG***	***RICTOR***	***SPOP***	***UBE2K***
*ADAMTSL1*	***BCL9***	***CD19***	***CTNNA1***	***ERCC1***	***FGFR2***	***HSP90AB1***	***KMT2D***	***MUC16***	***PAX8***	***PRKCI***	***RNF43***	***SRC***	***UBR5***
*ADGRA2*	***BCOR***	***CD274***	***CTNNB1***	***ERCC2***	***FGFR3***	***HSPA4***	***KRAS***	***MUC4***	***PBRM1***	***PRKCQ***	***ROS1***	***STAG2***	***UGT1A1*** *
ADH1C*	***BIRC2***	***CD58***	***CUL3***	***ERCC3***	***FGFR4***	***HSPA5***	***LCK***	***MUC6***	***PDCD1***	***PRKDC***	***RPPH1***	***STAT3***	***USH2A***
*AKT1*	***BIRC3***	***CD70***	***CYLD***	***ERCC4***	***FH***	***IDH1***	***LIG1***	***MUTYH***	***PDCD1LG2***	***PRKN***	***RPTOR***	***STK11***	***VDR*** *
*AKT2*	***BLM***	***CD79A***	CYP1A1*	***ERCC5***	***FLCN***	***IDH2***	***LIG3***	***MYC***	***PDGFRA***	***PSMB8***	***RUNX1***	***SUFU***	***VEGFA***
*AKT3*	***BMPR1A***	***CD79B***	CYP2B6*	***ERG***	***FLT1***	IFNL3*	***LMO1***	***MYCL***	***PDGFRB***	***PSMB9***	***RUNX1T1***	***SYK***	***VEGFB***
ALDH1A1*	***BRAF***	***CDC73***	CYP2C19*	***ESR1***	***FLT3***	***IGF1***	***LRP1B***	***MYCN***	***PDIA3***	***PSME1***	***RXRA***	***SYNE1***	***VHL***
*ALK*	***BRCA1***	***CDH1***	CYP2C8*	***ESR2***	***FLT4***	***IGF1R***	***LYN***	***MYD88***	***PGF***	***PSME2***	***SDHA***	***TAF1***	***WT1***
*AMER1*	***BRCA2***	***CDK1***	***CYP2D6***	***ETV1***	***FOXL2***	***IGF2***	***MALT1***	NAT2*	***PHOX2B***	***PSME3***	***SDHB***	***TAP1***	***XIAP***
*APC*	***BRD4***	***CDK12***	CYP2E1*	***ETV4***	***FOXP1***	***IKBKB***	***MAP2K1***	***NBN***	***PIK3C2B***	***PTCH1***	***SDHC***	***TAP2***	***XPO1***
*AR*	***BRIP1***	***CDK2***	CYP3A4*	***EZH2***	***FRG1***	***IKBKE***	***MAP2K2***	***NEFH***	***PIK3C2G***	***PTEN***	***SDHD***	***TAPBP***	***XRCC2***
*ARAF*	***BTG1***	***CDK4***	CYP3A5*	***FAM46C***	***FUBP1***	***IKZF1***	***MAP2K4***	***NF1***	***PIK3C3***	***PTGS2***	***SERPINB3***	***TBX3***	***ZNF217***
*ARID1A*	BTG2*	***CDK5***	***DAXX***	***FANCA***	***GATA1***	***IL6***	***MAP3K1***	***NF2***	***PIK3CA***	***PTPN11***	***SERPINB4***	***TEK***	
*ARID1B*	***BTK***	***CDK6***	***DCUN1D1***	***FANCC***	***GATA2***	***IL7R***	***MAP3K7***	***NFE2L2***	***PIK3CB***	***PTPRD***	***SETD2***	***TERT***	
*ARID2*	***BUB1B***	***CDK7***	***DDR2***	***FANCD2***	***GATA3***	***INPP4B***	***MAPK1***	***NFKB1***	***PIK3CD***	***PTPRT***	***SF3B1***	***TET1***	
*ASXL1*	***CALR***	***CDK8***	***DICER1***	***FANCE***	***GNA11***	***INSR***	***MAPK3***	***NFKBIA***	***PIK3CG***	***RAC1***	***SGK1***	***TET2***	
*ATM*	***CANX***	***CDK9***	***DNMT3A***	***FANCF***	***GNA13***	***IRF4***	***MAX***	***NKX2-1***	***PIK3R1***	***RAD50***	***SH2D1A***	***TGFBR2***	
*ATR*	***CARD11***	***CDKN1A***	***DOT1L***	***FANCG***	***GNAQ***	***IRS1***	***MCL1***	***NOTCH1***	***PIK3R2***	***RAD51***	SLC19A1*	TMSB4X*	
*ATRX*	***CASP8***	***CDKN1B***	***DPYD***	***FANCL***	***GNAS***	***IRS2***	***MDM2***	***NOTCH2***	***PIK3R3***	***RAD51B***	***SLC22A2***	***TNF***	
*AURKA*	***CBFB***	***CDKN2A***	***DTX1***	***FAS***	***GREM1***	***JAK1***	***MDM4***	***NOTCH3***	***PIM1***	***RAD51C***	***SLCO1B1***	***TNFAIP3***	

* Analysis of copy number alteration not available.

**Table 2:** Fusion genes of ACTOnco^®+^ assay. ACTOnco^®+^ assay identifies 31 fusion genes from RNA.

*ABL1*	*ALK*	*BCR*	*BRAF*	*CD74*	*ERG*	*ESR1*	*ETV1*	*ETV4*	*ETV5*
*ETV6*	***EZR***	***FGFR1***	***FGFR2***	***FGFR3***	***KMT2A (MLL***)	***MET***	***NRG1***	***NTRK1***	***NTRK2***
*NTRK3*	***NUTM1***	***PDGFRA***	***PDGFRB***	***RARA***	***RET***	***ROS1***	***RSPO2***	***SDC4***	***SLC34A2***
*TMPRSS2*									

**Figure 2.**
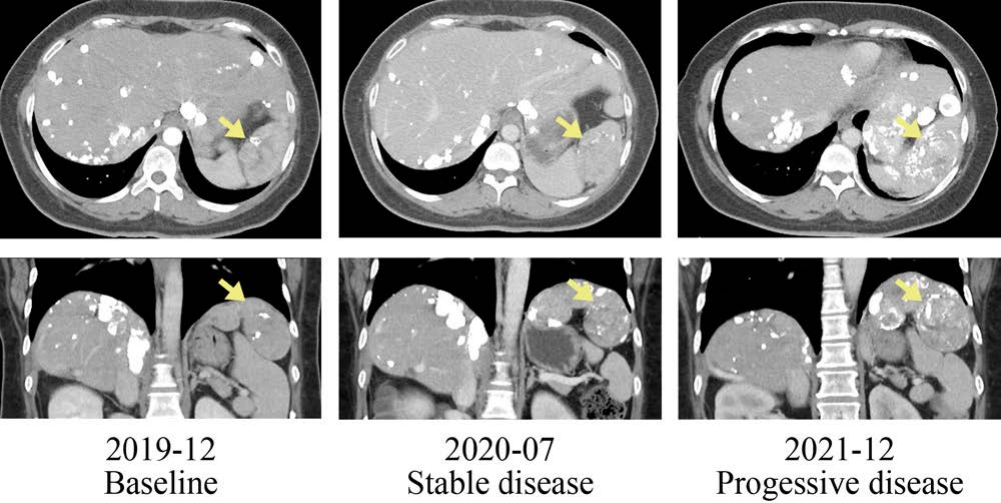
Computerized tomography scan before and after treatment.

**Figure 3.**
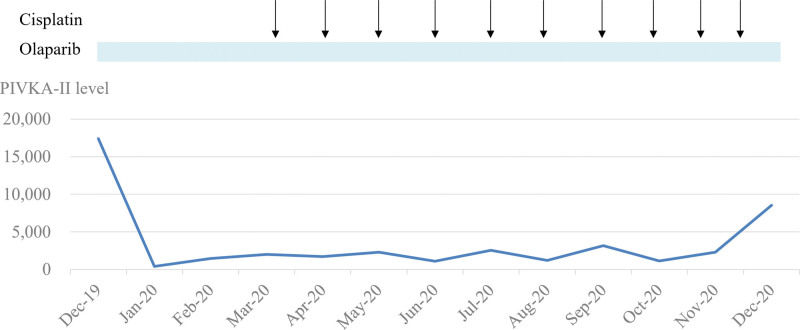
The dynamic change of tumor marker level (PIVKA-II).

Given *FANCA* belongs to DDR genes, the inactivation evoked the idea of using PARP inhibitor and cisplatin. Therefore, the patient started to use olaparib combined with low-dose cisplatin (30 mg/m^2^, every 4 weeks) therapy in December 2019. Significant reduction in the tumor marker level in 1 month (PIVKA-II from 17,395 to 411 ng/dL) and follow-up CT scan showed stable disease (Figures 2 and 3). The patient experienced grade 1 nausea without other severe adverse events during the cancer treatment. Her tumor did not progress until December 2020 with a progression-free survival of 12 months.

## 3. Discussion

The case with heavily treated, metastatic, *FANCA*-mutated HCC, had stable disease to the 12-month combination treatment of olaparib and low-dose cisplatin. Our experience with this case suggested that PARP inhibitor may be a potential therapeutic option for *FANCA* mutation; PARP inhibitor combined with cisplatin may lead to synergistic efficacy with tolerable toxicity; and DDR gene mutation may respond to PARP inhibitor in HCC.

The response for *FANCA* mutation to PARP inhibitor is not clear because of limited data. In a phase 2 clinical trial (TRITON2), 2 cases of metastatic castration-resistant prostate cancer patients with *FANCA* homozygous deletion responded to rucaparib (1 partial response and 1 stable disease).^[9]^ Another phase 2 study of olaparib for patients with metastatic castration-resistant prostate cancer, revealed 3 patients with homozygous deletion of *FANCA*, 1 of which had partial response.^[10]^ In yet another phase 2 study (TBCRC 048), metastatic breast cancer demonstrated 1 case of somatic *FANCA* mutation achieved stable disease with olaparib treatment.^[11]^ A study enrolling high-grade serous ovarian cancer harboring DDR gene mutation, with the exception of BRCA, showed better response to PARP inhibitors compared with those harboring wild-type DDR gene. *FANCA* mutation is presented in 1 patient of the DDR mutation group.^[12]^ Based on our literature review, *FANCA* mutation is rare among cancers. Although several reports showed the *FANCA* mutation is sensitive to the treatment of PRAP inhibitor, the role of PARP inhibitor in *FANCA* mutation is still controversial so far.

Genomic instability score (GIS) is calculated based on the results of homologous recombination repair mutation to predict the efficacy of PAPR inhibitor. In a phase 3 PAOLA-1/ENGOT-ov25 (NCT02477644) trial, GIS ≥ 42 was found to predict better PARP inhibitor efficacy. However, the mutation in *FANCA* gene present with a median GIS score of <42. This may explain the variable response of *FANCA* mutation to PARP inhibitor.^[13]^

Homologous recombination deficiency was known to render high response to the platinum agent that cross-link DNA strands leading to cell apoptosis. In addition, PARP inhibitor is being actively investigated with promising results in platinum-sensitive recurrent ovarian cancer. These provided a rationale to combine platinum agent with PARP inhibitor for the treatment of cancers with loss-of-function mutations in DDR genes. In fact, this combination has been found to improve progression-free survival in ovarian and breast cancers as opposed to those receiving chemotherapy alone.^[14]^ Therefore, a phase 2 clinical trial to explore efficacy and safety of olaparib in combination with carboplatin and paclitaxel in ovarian cancer is ongoing [NCT01081951]. In this case, olaparib combined with a relative low dose of cisplatin showed durable response, presumably a favorable synergistic effect.

This report has several limitations. First, NGS data was obtained from tumor tissue. Therefore, whether the mutation is somatic or germline is unknown. Second, ACTOnco^®+^ was used for the NGS testing that provided a panel of 440 oncogenes, making the genes outside the panel and the score of genomic instability unavailable.

## 4. Conclusion

In conclusion, we report the first case of *FANCA*-mutated HCC that responded well to olaparib and low-dose cisplatin. This addressed the potential therapeutic role of DDR gene mutation in HCC and the possible synergistic effect of PARP inhibitor and cisplatin. These findings highlight areas where further investigation and effort are needed.

## Acknowledgments

The author wishes to acknowledge Tan Kien Thiam for the support of genomic analysis.

## Author contributions

Conceived and designed the experiments: Y-HL, S-CC.

Performed the experiments: K-CT.

Analyzed the data: K-CT, S-CC.

Contributed reagents/materials/analysis tools: Y-HL, K-CT, S-CC.

Contributed to the writing of the manuscript: Y-HL, K-CT, S-CC.

**Conceptualization:** San-Chi Chen, Yi-Hsuan Lai.

**Data curation:** San-Chi Chen, Yi-Hsuan Lai.

**Formal analysis:** Kai-Che Tung, San-Chi Chen, Yi-Hsuan Lai.

**Methodology:** Kai-Che Tung.

**Supervision:** San-Chi Chen.

**Writing – original draft:** Kai-Che Tung, Yi-Hsuan Lai.

**Writing – review & editing:** San-Chi Chen.
